# Supplementary material for: MSH6/2 and PD-L1 Expressions Are Associated with Tumor Growth and Invasiveness in Silent Pituitary Adenoma Subtypes
Source: Int J Mol Sci. 2020 Apr 18;21(8):2831. doi: 10.3390/ijms21082831 (PMC7215962; doi:10.3390/ijms21082831)
Supplement: Supplementary file 1 [file ijms-21-02831-s001.pdf]

Supplemental Figure 1

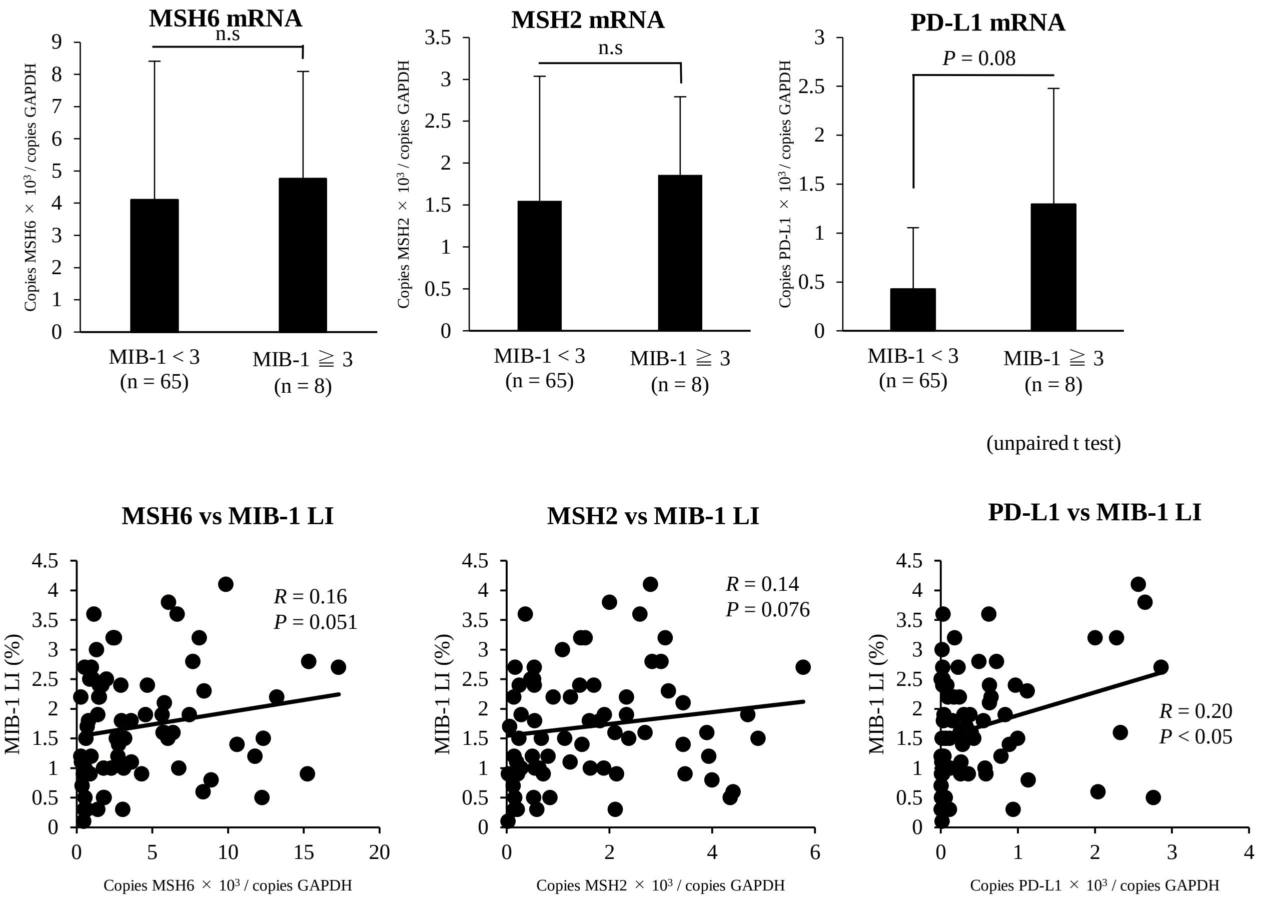

Supplemental Figure 2

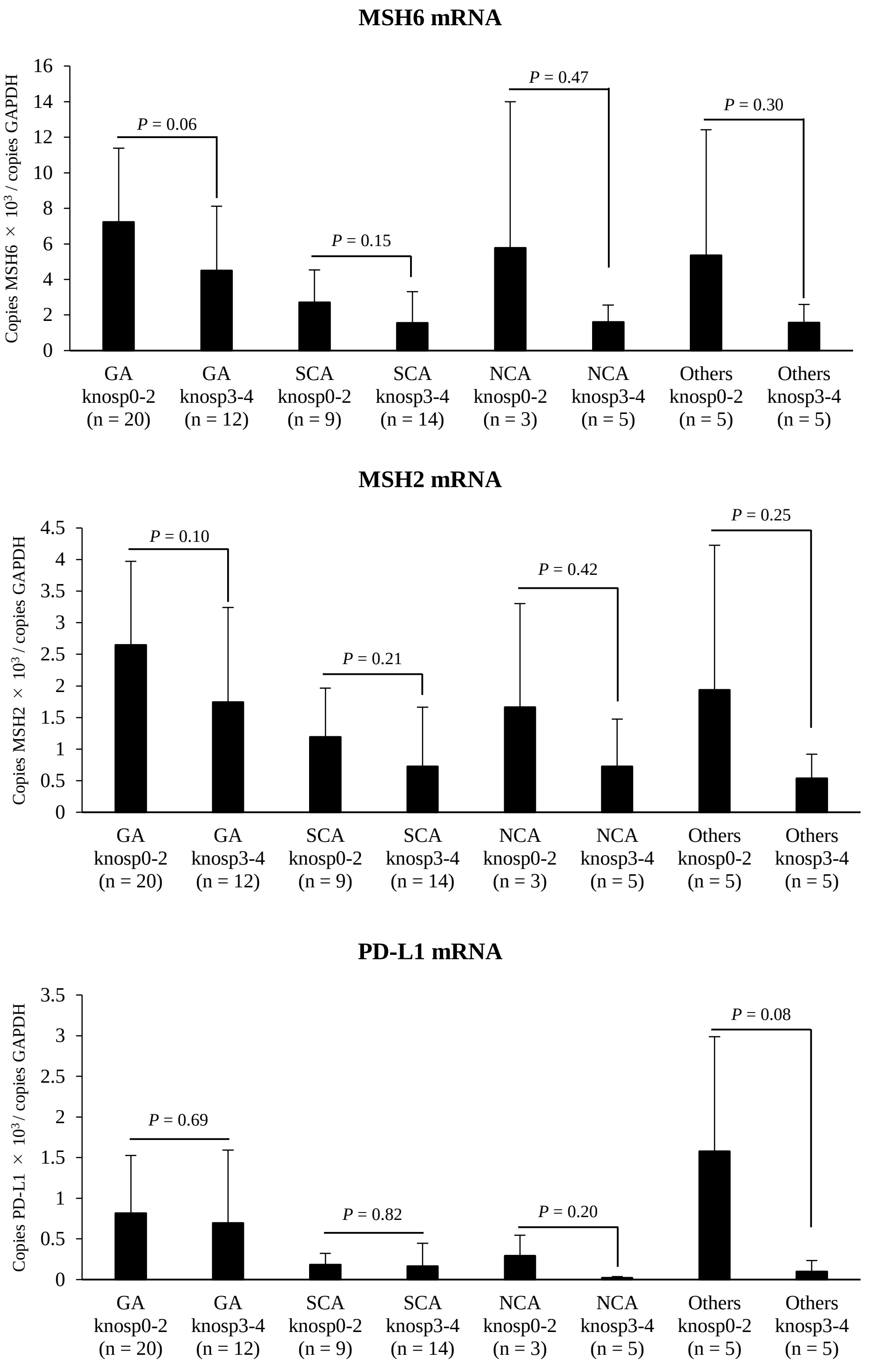

Supplemental Figure 3

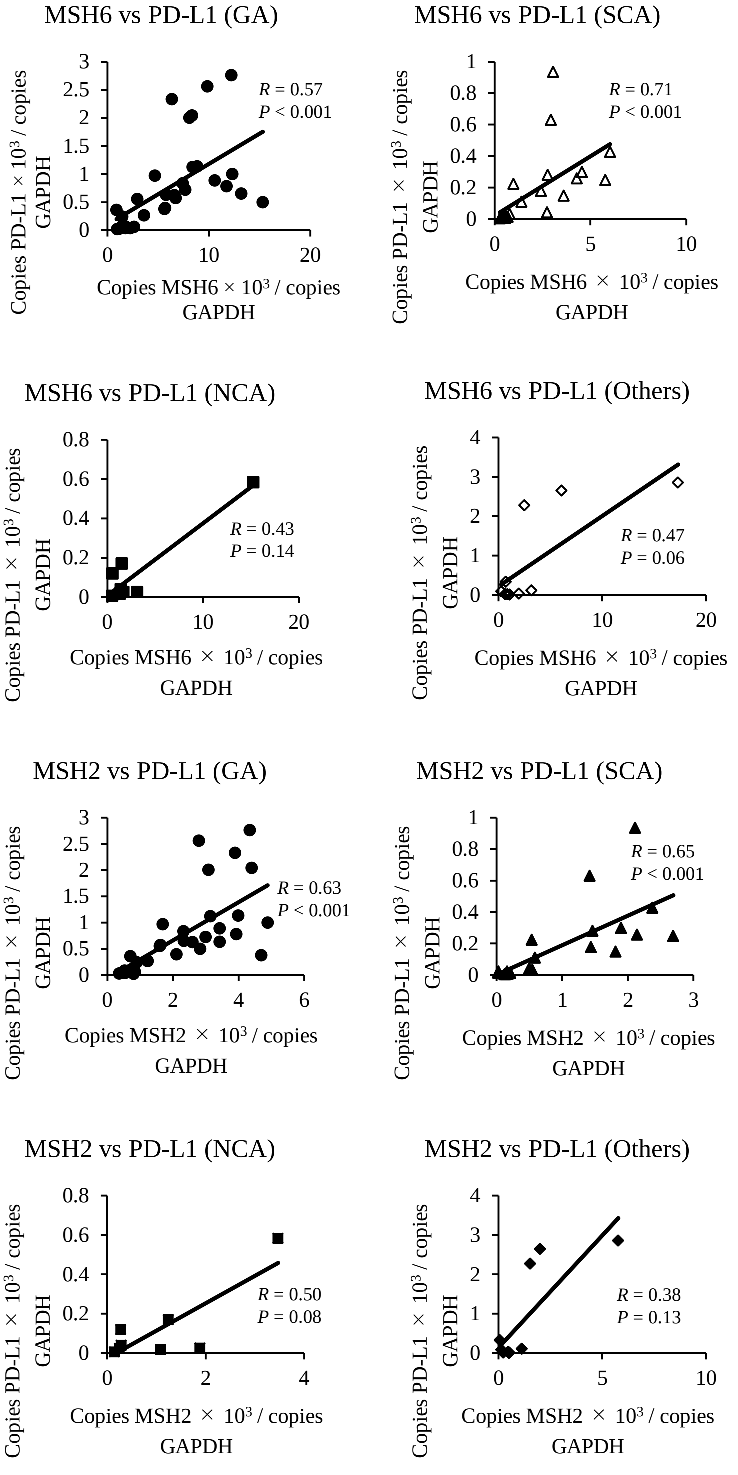

Supplemental Table1

Case Number	sex	age	Clinical phenotype	Pathological phenotype	micro/macro	MIB-1 LI(%)	knoso grade	visual disturbance	reoperation	Immunohistochemistry								
										ACTH	FSH	LH	GH	PRL	TSH	TPIT	SF1	PIT1
1	male	65	Nonfunctioning adenoma	Gonadotroph adenoma	macro	0.5	0	+	-	-	+	+	-	-	-	n.a	n.a	n.a
2	male	66	Nonfunctioning adenoma	Gonadotroph adenoma	macro	2.8	0	+	-	-	-	-	-	-	-	n.a	+	n.a
3	male	68	Nonfunctioning adenoma	Gonadotroph adenoma	macro	1	1	+	-	-	-	-	-	-	-	n.a	+	n.a
4	male	41	Nonfunctioning adenoma	Gonadotroph adenoma	macro	1.4	1	+	-	-	+	-	-	-	-	n.a	n.a	n.a
5	male	36	Nonfunctioning adenoma	Gonadotroph adenoma	macro	1.1	1	+	-	-	+	-	-	-	-	n.a	+	n.a
6	male	35	Nonfunctioning adenoma	Gonadotroph adenoma	macro	2.2	1	+	-	-	-	-	-	-	-	n.a	+	n.a
7	female	80	Nonfunctioning adenoma	Gonadotroph adenoma	macro	1.6	1	+	-	-	-	+	-	-	-	n.a	+	-
8	female	62	Nonfunctioning adenoma	Gonadotroph adenoma	macro	0.8	1	+	-	-	-	-	-	-	-	n.a	+	-
9	male	53	Nonfunctioning adenoma	Gonadotroph adenoma	macro	2.2	1	+	-	-	-	-	-	-	-	n.a	+	-
10	male	56	Nonfunctioning adenoma	Gonadotroph adenoma	macro	0.9	1	+	-	-	+	-	-	-	-	n.a	n.a	n.a
11	male	43	Nonfunctioning adenoma	Gonadotroph adenoma	macro	1.2	1	-	-	-	+	-	-	-	-	n.a	+	n.a
12	male	43	Nonfunctioning adenoma	Gonadotroph adenoma	macro	1	1	-	-	-	+	-	-	-	-	n.a	+	n.a
13	female	41	Nonfunctioning adenoma	Gonadotroph adenoma	macro	0.6	2	+	-	-	+	-	-	-	-	n.a	n.a	n.a
14	female	21	Nonfunctioning adenoma	Gonadotroph adenoma	macro	3.2	2	+	-	-	-	-	-	-	-	n.a	+	-
15	female	62	Nonfunctioning adenoma	Gonadotroph adenoma	macro	1.2	2	+	-	-	+	-	-	-	-	n.a	n.a	n.a
16	male	69	Nonfunctioning adenoma	Gonadotroph adenoma	macro	1.9	2	-	-	-	+	-	-	-	-	n.a	n.a	n.a
17	male	56	Nonfunctioning adenoma	Gonadotroph adenoma	macro	1.9	2	-	-	-	-	-	-	-	-	n.a	+	n.a
18	female	62	Nonfunctioning adenoma	Gonadotroph adenoma	macro	2.3	2	+	-	-	-	-	-	-	-	n.a	+	n.a
19	female	36	Nonfunctioning adenoma	Gonadotroph adenoma	macro	2.8	2	+	-	-	-	-	-	-	-	n.a	+	-
20	female	54	Nonfunctioning adenoma	Gonadotroph adenoma	macro	2.1	2	+	-	-	-	-	-	-	-	n.a	+	-
21	female	53	Nonfunctioning adenoma	Gonadotroph adenoma	macro	1.6	3	-	-	-	+	+	-	-	-	n.a	n.a	n.a
22	male	60	Nonfunctioning adenoma	Gonadotroph adenoma	macro	2.4	3	+	-	-	-	-	-	-	-	n.a	+	n.a
23	male	50	Nonfunctioning adenoma	Gonadotroph adenoma	macro	1.5	3	+	-	-	+	-	-	-	-	n.a	+	-
24	male	53	Nonfunctioning adenoma	Gonadotroph adenoma	macro	3.6	3	+	-	-	+	-	-	-	-	n.a	+	-
25	male	41	Nonfunctioning adenoma	Gonadotroph adenoma	macro	1.8	3	+	-	-	-	-	-	-	-	n.a	+	-
26	male	44	Nonfunctioning adenoma	Gonadotroph adenoma	macro	0.5	3	+	-		+	-	-	-	-	n.a	+	n.a
27	female	62	Nonfunctioning adenoma	Gonadotroph adenoma	macro	0.5	3	+	-	-	+	-	-	-	-	n.a	n.a	n.a
28	male	50	Nonfunctioning adenoma	Gonadotroph adenoma	macro	1.5	3	+	-	-	+	-	-	-	-	n.a	+	-
29	male	53	Nonfunctioning adenoma	Gonadotroph adenoma	macro	3.6	3	+	-	-	+	-	-	-	-	n.a	+	-
30	male	71	Nonfunctioning adenoma	Gonadotroph adenoma	macro	4.1	4	+	-	-	-	-	-	-	-	n.a	+	-
31	male	45	Nonfunctioning adenoma	Gonadotroph adenoma	macro	1	4	+	-		+	-	-	-	-	n.a	+	n.a
32	male	66	Nonfunctioning adenoma	Gonadotroph adenoma	macro	2.4	4	+	-	-	+	-	-	-	-	n.a	+	n.a
33	male	60	Nonfunctioning adenoma	null cell adenoma	macro	0.9	1	-	-	-	-	-	-	-	-	-	-	-
34	male	73	Nonfunctioning adenoma	null cell adenoma	macro	1	1	-	-	-	-	-	-	-	-	-	-	-
35	female	53	Nonfunctioning adenoma	null cell adenoma	macro	2.2	2	-	-	-	-	-	-	-	-	-	-	-
36	male	53	Nonfunctioning adenoma	null cell adenoma	macro	1	3	+	-	-	-	-	-	-	-	-	-	-
37	male	76	Nonfunctioning adenoma	null cell adenoma	macro	2.4	3	+	-	-	-	-	-	-	-	-	-	-
38	male	45	Nonfunctioning adenoma	null cell adenoma	macro	3	4	-	-	-	-	-	-	-	-	-	-	-
39	male	51	Nonfunctioning adenoma	null cell adenoma	macro	1.9	4	-	-	-	-	-	-	-	-	-	-	-
40	female	52	Nonfunctioning adenoma	null cell adenoma	macro	0.3	4	-	-	-	-	-	-	-	-	-	-	-
41	female	40	Nonfunctioning adenoma	Silent corticotroph adenoma	macro	1.5	1	+	-	+	-	-	-	-	-	n.a	-	-
42	male	61	Nonfunctioning adenoma	Silent corticotroph adenoma	macro	1.9	1	+	-	+	-	-	-	-	-	n.a	-	-
43	female	50	Nonfunctioning adenoma	Silent corticotroph adenoma	macro	1.8	1	+	-	+	-	-	-	-	-	n.a	-	-
44	female	30	Nonfunctioning adenoma	Silent corticotroph adenoma	macro	2.7	1	+	-	+	-	-	-	-	-	n.a	-	-
45	female	30	Nonfunctioning adenoma	Silent corticotroph adenoma	macro	2.7	1	+	-	+	-	-	-	-	-	n.a	-	-
46	male	68	Nonfunctioning adenoma	Silent corticotroph adenoma	macro	1.4	2	-	-	+	-	-	-	-	-	n.a	-	-
47	female	50	Nonfunctioning adenoma	Silent corticotroph adenoma	macro	1.8	2	-	-	+	-	-	-	-	-	n.a	-	-
48	female	39	Nonfunctioning adenoma	Silent corticotroph adenoma	macro	3.2	2	-	-	+	-	-	-	-	-	n.a	-	-
49	female	48	Nonfunctioning adenoma	Silent corticotroph adenoma	macro	1.2	2	+	-	+	-	-	-	-	-	+	-	-
50	female	72	Nonfunctioning adenoma	Silent corticotroph adenoma	macro	0.3	3	+	-	+	-	-	-	-	-	n.a	-	-
51	female	43	Nonfunctioning adenoma	Silent corticotroph adenoma	macro	0.9	3	+	-	+	-	-	-	-	-	n.a	-	-
52	female	52	Nonfunctioning adenoma	Silent corticotroph adenoma	macro	0.9	3	-	-	+	-	-	-	-	-	n.a	-	-
53	female	72	Nonfunctioning adenoma	Silent corticotroph adenoma	macro	0.3	3	+	-	+	-	-	-	-	-	n.a	-	-
54	female	45	Nonfunctioning adenoma	Silent corticotroph adenoma	macro	0.9	3	-	-	+	-	-	-	-	-	n.a	-	-
55	female	56	Nonfunctioning adenoma	Silent corticotroph adenoma	macro	0.7	3	+	-	+	-	-	-	-	-	n.a	-	-
56	female	65	Nonfunctioning adenoma	Silent corticotroph adenoma	macro	2.4	4	+	-	+	-	-	-	-	-	n.a	-	-
57	female	53	Nonfunctioning adenoma	Silent corticotroph adenoma	macro	1.6	4	+	-	+	-	-	-	-	-	n.a	-	-
58	female	52	Nonfunctioning adenoma	Silent corticotroph adenoma	macro	0.3	4	-	-	+	-	-	-	-	-	n.a	-	-
59	male	44	Nonfunctioning adenoma	Silent corticotroph adenoma	macro	1.1	4	+	-	+	-	-	-	-	-	+	-	-
60	female	47	Nonfunctioning adenoma	Silent corticotroph adenoma	macro	1.2	4	+	-	+	-	-	-	-	-	n.a	n.a	n.a
61	female	60	Nonfunctioning adenoma	Silent corticotroph adenoma	macro	0.5	4	-	-	+	-	-	-	-	-	n.a	n.a	n.a
62	female	63	Nonfunctioning adenoma	Silent corticotroph adenoma	macro	0.9	4	+	-	+	-	-	-	-	-	n.a	-	-
63	male	49	Nonfunctioning adenoma	Silent corticotroph adenoma	macro	0.1	4	+	-	+	-	-	-	-	-	n.a	-	-
64	male	31	Nonfunctioning adenoma	Silent lactotroph adenoma	macro	3.8	1	-	-	-	-	-	-	+	-	n.a	n.a	n.a
65	female	38	Nonfunctioning adenoma	Silent lactotroph adenoma	macro	1.5	2	+	-	-	-	-	-	+	-	-	-	+
66	male	46	Nonfunctioning adenoma	Silent lactotroph adenoma	macro	2.5	3	-	-	-	-	-	-	+	-	-	-	+
67	female	74	Nonfunctioning adenoma	Silent lactotroph adenoma	macro	2.5	3	+	-	-	-	-	-	+	-	-	-	+
68	female	37	Nonfunctioning adenoma	silent plurihormonal adenoma(PRL, GH)	macro	2.7	0	-	-	-	-	-	+	+	-	n.a	-	+
69	male	43	Nonfunctioning adenoma	silent plurihormonal adenoma(PRL, GH)	macro	2.2	2	+	-	-	-	-	-	+	+	n.a	-	+
70	male	80	Nonfunctioning adenoma	silent plurihormonal adenoma(PRL, GH)	macro	1.5	3	+	-	-	-	-	+	+	+	n.a	-	+
71	female	40	Nonfunctioning adenoma	silent plurihormonal adenoma(PRL, GH)	macro	2.5	3	+	-	-	-	-	+	+	-	n.a	-	+
72	female	21	Nonfunctioning adenoma	Silent thyrotroph adenoma	macro	3.2	1	-	-	-	-	-	-	-	+	n.a	-	+
73	female	58	Nonfunctioning adenoma	Silent thyrotroph adenoma	macro	1.7	3	-	-	-	-	-	-	-	+	-	-	+

Supplemental Table 2A

qRT-PCR primers used to measure mRNA expression (human *MSH2* , *MSH6* , *PD-L1* , and *GAPDH*).

human		Primers	length	PCR products size
MSH2	Forward	5'-ACCAGCAGCAAAGAAGTGCT	20mer	86bp
	Reverse	5'-AGGGCATTTGTTTCACCTTG	20mer	
MSH6	Forward	5'-CATGCGGCGACTGTTCTAT	19mer	145bp
	Reverse	5'-CAGAATTACTGGGCGACACA	20mer	
PD-L1 (CD274)	Forward	5'-TGCAGGGCATTCCAGAAAGA	20mer	100bp
	Reverse	5'-TAGGTCCTTGGGAACCGTGA	20mer	
GAPDH	Forward	5'-GAAGGTGAAGGTCGGAGTCA	20mer	226bp
	Reverse	5'-GAAGATGGTGATGGGATTTC	20mer	

Supplemental Table 2B

qRT-PCR primers used to measure mRNA expression (mouse *PD-L1* and *GAPDH*).

mouse		Primers	length	PCR products size
PD-L1 (CD274)	Forward	5'-GCAACACATCCTCCACAGAA	20mer	139bp
	Reverse	5'-CTTCAACGCCACATTTCTCC	20mer	
GAPDH	Forward	5'-GAAGGTGAAGGTCGGAGTCA	20mer	171bp
	Reverse	5'-GAAGATGGTGATGGGATTTC	20mer	

Supplemental Table 2C

qRT-PCR primers used to measure mRNA expression (rat *PD-L1* and *GAPDH*).

rat		Primers	length	PCR products size
PD-L1 (CD274)	Forward	5'-TGCAGGGCATTCCAGAAAGA	20mer	100bp
	Reverse	5'-TAGGTCCTTGGGAACCGTGA	20mer	
GAPDH	Forward	5'-GAAGGTGAAGGTCGGAGTCA	20mer	226bp
	Reverse	5'-GAAGATGGTGATGGGATTTC	20mer	
